# The impact of neoadjuvant immunochemotherapy on the prognosis of locally advanced oral carcinoma

**DOI:** 10.3389/froh.2026.1744191

**Published:** 2026-03-17

**Authors:** Yujie Ke, Zhicong Hong, Limei Guan, Xianyang Luo, Zhenhong Ye

**Affiliations:** 1 Department of Otolaryngology Head and Neck Surgery, The First Affiliated Hospital of Xiamen University, School of Medicine, Xiamen University, Xiamen, China; 2 Department of Reproductive Medicine, The Xiamen Maternity and Child Healthcare Hospital, School of Medicine, Xiamen University, Xiamen, China

**Keywords:** disease-free survival, immune checkpoint inhibitors, neoadjuvant immunochemotherapy, oral squamous cell carcinomas, overall survival

## Abstract

**Objective:**

This study aimed to investigate the effect of preoperative neoadjuvant immunochemotherapy (NAICT) on the prognosis of patients with locally advanced oral squamous cell carcinomas (OSCC), thereby providing evidence-based guidance for the clinical management of OSCC.

**Methods:**

We conducted a retrospective cohort study of OSCC cases treated at our institution between 2017 and 2022. Kaplan–Meier survival curves, along with univariate and multivariate Cox regression analyses, were employed to identify independent factors influencing 3-year overall survival (OS) and disease-free survival (DFS) rates.

**Results:**

A total of 158 patients with locally advanced OSCC were included. 119 patients received simple surgical treatment, 15 patients underwent neoadjuvant chemotherapy (NAC) followed by surgery and 24 patients underwent NAICT followed by surgery. Multivariate analysis identified differentiation (*P* = 0.041), negative margins (*P* = 0.043), N stage (*P* = 0.046), treatment strategy (*P* = 0.016), postoperative recurrence (*P* < 0.001), and postoperative metastasis (*P* = 0.044) as independent factors influencing 3-year OS. For 3-year DFS, the independent factors included negative margins (*P* = 0.017), N stage (*P* = 0.006), treatment strategy (*P* = 0.004), postoperative recurrence (*P* < 0.001), and post-operative metastasis (*P* < 0.001). Compared to direct surgery, surgery following NAC showed no significant difference in 3-year OS (53.3% vs. 57.1%, *P* = 0.701) and 3-year DFS (33.3% vs. 52.9%, *P* = 0.099). Compared to direct surgery, preoperative NAICT significantly improved 3-year OS (82.4% vs. 57.1%, *P* = 0.004) and 3-year DFS (83.3% vs. 52.9%, *P* = 0.007). Furthermore, compared to surgery following NAC, preoperative NAICT was associated with a significantly improved 3-year OS (82.4% vs. 53.3%, *P* = 0.03) and 3-year DFS (83.3% vs. 33.3%, *P* = 0.001). Treatment-related adverse events occurred in 70.8% of patients receiving NAICT. All events were grade 1–2 in severity and resolved following treatment.

**Conclusion:**

Compared to direct surgery or NAC followed by surgery, preoperative NAICT demonstrated a more favorable impact on the prognosis of patients with locally advanced OSCC, while exhibiting manageable treatment-related adverse effects.

## 1 Introduction

Worldwide, the incidence of oral squamous cell carcinomas (OSCC) poses a substantial and mounting challenge to public health. These cancers are not only the predominant malignancy of the oral cavity but also rank among the world's top 10 most common cancers (1). According to the latest global cancer statistics report, in 2022, there were more than 380,000 new cases and over 180,000 deaths from OSCC. Despite considerable efforts in treating OSCC, their five-year survival rates remain suboptimal (2). The survival outcomes differ dramatically for those with localized OSCC vs. regional or distant metastases. The 5-year survival rate for localized disease is about 80%, in contrast to a 40% to 50% and less than 10% 5-year survival for those with regional and distant metastases, respectively (3–6). Hence, it is imperative to devise more effective therapeutic strategies to improve the prognosis of locally advanced OSCC.

The tumor microenvironment in head and neck squamous cell carcinoma (HNSCC) is uniquely adapted for immune evasion, characterized by tumor-mediated co-option of immune cells. This immunosuppressive milieu critically involved the Programmed Death-1 (PD-1) pathway: Binding of the immune checkpoint receptor PD-1 to its ligand PD-L1 (frequently overexpressed by tumor cells) inhibits T-cell function, enabling escape from immune surveillance. Immune checkpoint inhibitors (ICIs) target the PD-1/PD-L1 axis to reverse T-cell exhaustion, enabling restored tumor immune surveillance and consequent growth suppression (7, 8). Clinical evidence confirms survival benefits from ICIs in both first- and second-line treatment of recurrent/metastatic HNSCC (9–12). Compared to standard chemotherapy, the anti-PD-1 inhibitor Nivolumab achieved a 32% reduction in the hazard ratio for mortality and improved the 2-year overall survival (OS) rate nearly threefold to 16.9% in patients with recurrent/metastatic HNSCC (13, 14). Multiple trials demonstrate that neoadjuvant immunochemotherapy (NAICT) significantly prolongs survival and elevates pathological complete response (PCR) or major pathologic response (MPR) rates across solid tumors (15–20). In a phase II trial for locally advanced HNSCC, the PCR rate reached 37%, and the MPR rate was 74.1% (21). Recently, the KEYNOTE-689 phase III trial have reported that the addition of neoadjuvant and adjuvant pembrolizumab to standard care significantly improved event-free survival among participants with locally advanced HNSCC (22).

Although NAICT demonstrates considerable potential in HNSCC treatment, its impact on the prognosis of locally advanced OSCC remains undetermined. Therefore, this study aims to assess its clinical impact on medium-term prognosis in locally advanced OSCC by comparing 3-year OS and disease-free survival (DFS) rates across established therapeutic approaches.

## 2 Methods

### 2.1 Patients

We retrospectively included patients with locally advanced OSCC at our institution from January 2017 to August 2022. Inclusion criteria: (1) Histologically or cytologically confirmed diagnosis of squamous cell carcinoma; (2) Patients receive one of the following treatment regimens: simple surgical resection, neoadjuvant chemotherapy (NAC) followed by surgery, or NAICT followed by surgery. Exclusion criteria: (1) Refused surgical treatment or unable to tolerate general anesthesia surgery; (2) Combined with other malignant tumors; (3) Distant metastases (M1); (4) Patients with American Joint Committee on Cancer (AJCC) stage I and II. 2161;. The study was conducted in accordance with the Declaration of Helsinki (as revised in 2013) and received approval from the Ethics Committee of the First Affiliated Hospital of Xiamen University. All patients provided written informed consent before treatment.

### 2.2 Treatment

Our institution's multidisciplinary team (MDT) reviewed each patient's condition to formulate a comprehensive treatment plan. The treatment options include surgical resection, preoperative NAC, and preoperative NAICT. Surgical resection refers to wide local excision of the primary lesion and functional cervical lymph node dissection as needed. Preoperative NAC involves the local intra-arterial infusion of chemotherapeutic agents via an interventional approach 10–14 days prior to surgery. The regimen consists of: Cisplatin 60–80 mg and Pirarubicin 20–30 mg. Preoperative NAICT entails administering two cycles of immunotherapy combined with chemotherapy prior to surgery, with each cycle lasting 21 days. During the neoadjuvant therapy period, the patients received ICI (Pembrolizumab 200 mg; Tislelizumab 200 mg; Nivolumab 240 mg; Camrelizumab 200 mg) combined with albumin-bound paclitaxel 260 mg/m^2^ on day 1 and cisplatin 75 mg/m^2^ on day 1–3 (the total dose was administered in three equal injections), or carboplatin at a dose sufficient to achieve an area under the concentration-time curve of 5 mg/min/mL on day 1. Carboplatin dosing was determined using the Calvert formula: Dose (mg) = 5 × [Glomerular Filtration Rate (GFR, mL/min) + 25]. The GFR was estimated by the Cockcroft-Gault formula. For males: GFR (mL/min) = [(140 − age) × body weight (kg)]/[0.818 × serum creatinine (μmol/L)]. For females: GFR (mL/min) = 0.85 × [(140 − age) × body weight (kg)]/[0.818 × serum creatinine (μmol/L)]. Surgery was performed 3 to 4 weeks after NAICT. Prior to surgery, repeat computed tomography (CT), magnetic resonance imaging (MRI), or 18-fluorodexoyglucose positron emission tomography with computed tomography (18FDG-PET/CT) were conducted to assess the extent of the primary tumor and cervical lymph node involvement. The extent of surgical resection was discussed and determined by the MDT based on the post-treatment response to neoadjuvant therapy. Postoperatively, our MDT re-evaluated the patient's status. Based on the observation of adverse pathological features and in accordance with the National Comprehensive Cancer Network (NCCN) guidelines for oral cavity cancers, the decision was made regarding the indication for adjuvant radiotherapy or chemoradiotherapy. Specifically, patients exhibiting extra nodal extension or positive margins will receive adjuvant chemoradiotherapy. Adjuvant radiotherapy will be administered to patients with primary tumors classified as pT3 or pT4, nodal disease staged as pN2 or pN3, involvement of level IV or V lymph nodes, or the presence of perineural invasion, vascular invasion, or lymphatic invasion.

Patients underwent follow-up assessments at 3- to 6-month intervals postoperatively. Each evaluation included physical examination and diagnostic imaging (CT, MRI, or PET-CT). All participants were monitored for at least 3 years, with survival duration, tumor recurrence, and metastasis status documented through telephone consultations and outpatient clinical visits.

### 2.3 Evaluation of the efficacy of NAICT

All patients underwent CT, MRI, or PET/CT before and after treatment to delineate the extent of primary tumors and cervical lymph node metastases and evaluate response to NAICT. Tumor response was assessed 3 weeks following the final cycle of preoperative NAICT using Response Evaluation Criteria in Solid Tumors (RECIST) version 1.1 (23, 24), with outcomes categorized as complete response (CR), partial response (PR), stable disease (SD), or progressive disease (PD). In terms of pathological assessment, PCR was defined as the absence of residual tumor tissue in either primary lesions or cervical metastatic lymph nodes. MPR was defined as less than 10% residual tumor in the primary site upon pathological examination. Incomplete pathologic response (IPR) was defined as the presence of 10% or more viable tumor cells in the primary lesion. Radiological and pathologic responses were assessed by three radiologists and three pathologists, respectively, all blinded to group allocation, with the average of the three physicians’ assessments taken as the final result.

### 2.4 Assessment of adverse reactions

Follow-up data on adverse events were collected through outpatient visits and telephone consultations to monitor and record adverse events associated with preoperative NAICT. Throughout the treatment period, patients underwent weekly assessments of hematologic, hepatic, renal, and cardiac function, along with thyroid function assessments every three weeks. These events were graded using the Common Terminology Criteria for Adverse Events (CTCAE) version 5.0, which classifies adverse events into five levels including Grade 1: mild - mild symptoms that do not require treatment, Grade 2: moderate - moderate symptoms that may require treatment, Grade 3: severe - severe symptoms that require treatment, Grade 4: threat to life symptoms that are life-threatening and require urgent treatment, and Grade 5: death-adverse events resulting in death.

### 2.5 Statistical analysis

Descriptive statistics were used to analyze patient baseline characteristics. Survival estimates of all variables were generated using the Kaplan–Meier method, while the log-rank tests were used to compare the difference among the groups. Cox proportional hazards model (univariate analysis and multivariate analysis) assessed the independent prognostic factors associated with the survival. Variables significantly associated with survival in univariate analysis (*P* < 0.1) were included in the multivariate analysis. HR and 95% CI were used to show the degree of influence of each factor on OS or DFS. In order to control confounding and selection bias, a propensity score-weighted analysis was performed. Propensity score-weighted analysis balances the distribution of confounding factors between groups by assigning a recalculated weight to each patient. This process ensures that, after weighting, the groups achieve a fully balanced distribution across all measured confounders. To estimate the propensity scores, the covariates were used as main effects in a logistic regression model. Separate models were fitted for the comparison of NAICT plus surgery vs. surgery and the comparison of NAC plus surgery vs. surgery. This modeled the probability of a patient in the analysis population being treated with NAICT plus surgery or NAC plus surgery. The covariates significantly associated with survival in univariate analysis (*P* < 0.05) were included in the final propensity score model. The propensity scores were converted to inverse probability of treatment weights (IPTW) with NAICT plus surgery or NAC plus surgery patients having a weighting of 1. These IPTWs were then used in weighted survival analysis to adjust for differences in patient characteristics between treatments. All statistical analyses were performed using SPSS 24.0 or RStudio 4.5.1, and a value of 2-sided *P* < 0.05 was considered statistically significant.

### 2.6 Bias control

As a retrospective study, rigorous bias control is of paramount importance. In this study, selection bias was minimized by implementing stringent inclusion and exclusion criteria and maintaining a low lost-to-follow-up rate of only 3%. Information bias was mitigated through independent multi-observer reviews of imaging and pathological data. To address confounding bias, statistical adjustments were performed using multivariate analysis, propensity score matching, and inverse probability weighting. These methodological details have been comprehensively described in the preceding sections.

## 3 Results

### 3.1 Patient characteristics

**Figure 1:**
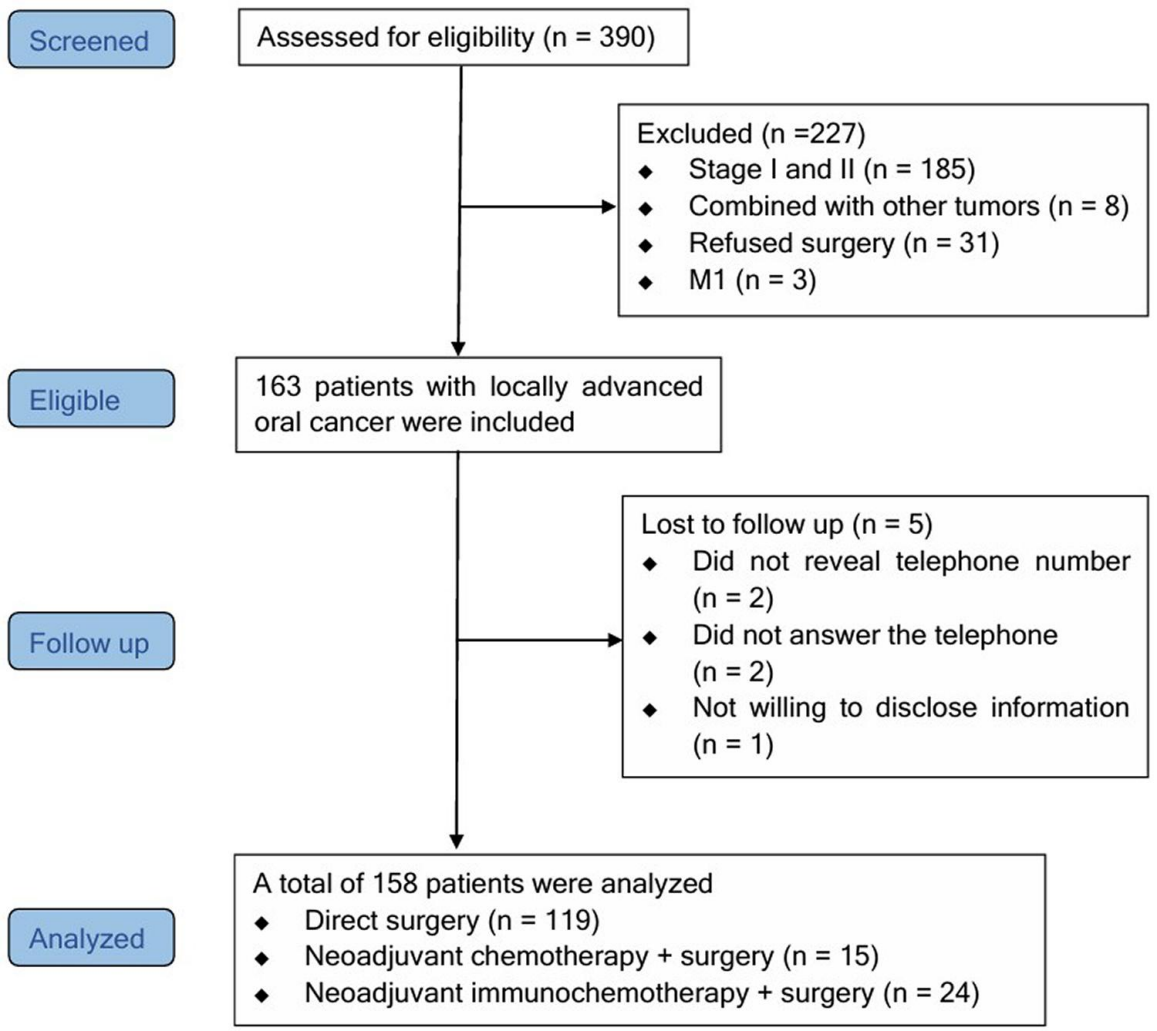
STROBE flow chart. STROBE, Strengthening the Reporting of Observational Studies in Epidemiology.

According to the inclusion criteria, 390 OSCC cases were enrolled. Based on the exclusion criteria, 227 cases were excluded, 5 cases were lost to follow up. Ultimately, 158 patients were included in the subsequent data analysis (Figure 1).

All patients’ median age was 60 years. Over half of the patients (*n* = 102, 64.6%) were male. Among all these primary sites, the tongue (50.6%) appeared to be the most common one, followed by cheek mucosa (17.7%) and the gum (12.7%). Moderately differentiated squamous cell carcinoma accounted for the highest proportion (72.2%). The patients with pathological T3 stage (38.0%), pathological N2 stage (39.2%) accounted for the vast majority. As for the treatment methods, 119 patients received simple surgical treatment, 15 patients underwent NAC followed by surgery and 24 patients underwent NAICT followed by surgery. Postoperative recurrence occurred in 41 patients and postoperative metastasis occurred in 4 patients (Table 1).

**Table 1:** Patient demographic data and clinical characteristics.

Characteristics	Total (*n* = 158)	Surgery (*n* = 119)	NAC (*n* = 15)	NAICT (*n* = 24)
	*N* (%)	*N* (%)	*N* (%)	*N* (%)
**Age (years)**				
≤40	7 (4.4)	6 (5.0)	0 (0)	1 (4.2)
41–50	24 (15.2)	19 (16.0)	5 (33.3)	0 (0)
51–60	49 (31.0)	31 (26.1)	8 (53.3)	10 (41.7)
61–70	48 (30.4)	39 (32.8)	1 (6.7)	8 (33.3)
71–80	25 (15.8)	19 (16.0)	1 (6.7)	5 (20.8)
81–90	5 (3.2)	5 (4.2)	0 (0)	0 (0)
**Sex**				
Male	102 (64.6)	72 (60.5)	14 (93.3)	16 (66.7)
Female	56 (35.4)	47 (39.5)	1 (6.7)	8 (33.3)
**Smoking history**				
Yes	61 (38.6)	39 (32.8)	9 (60.0)	13 (54.2)
No	97 (61.4)	80 (67.2)	6 (40.0)	11 (45.8)
**Alcohol history**				
Yes	44 (27.8)	28 (23.5)	8 (53.3)	8 (33.3)
No	114 (72.2)	91 (76.5)	7 (46.7)	16 (66.7)
**Tumor location**				
Tongue	80 (50.6)	60 (50.4)	11 (73.3)	9 (37.5)
Cheek mucosa	28 (17.7)	20 (16.8)	3 (20.0)	5 (20.8)
Floor of the mouth	17 (10.8)	12 (10.1)	1 (6.7)	4 (16.7)
Gum	20 (12.7)	17 (14.3)	0 (0)	3 (12.5)
Hard palate	7 (4.4)	4 (3.4)	0 (0)	3 (12.5)
Lip	6 (3.8)	6 (5.0)	0 (0)	0 (0)
**Differentiation**				
High	22 (13.9)	18 (15.1)	3 (20.0)	1 (4.2)
Middle	114 (72.2)	86 (72.3)	9 (60.0)	19 (79.2)
Low	22 (13.9)	15 (12.6)	3 (20.0)	4 (16.7)
**R0**				
Yes	152 (96.2)	115 (96.6)	15 (100.0)	22 (91.7)
No	6 (3.8)	4 (3.4)	0 (0)	2 (8.3)
**T stage**				
T1	10 (6.3)	9 (7.6)	0 (0)	1 (4.2)
T2	43 (27.2)	33 (27.7)	5 (33.3)	5 (20.8)
T3	60 (38.0)	46 (38.7)	9 (60.0)	5 (20.8)
T4	45 (28.5)	31 (26.1)	1 (6.7)	13 (54.2)
**N stage**				
N0	58 (36.7)	44 (37.0)	5 (33.3)	9 (37.5)
N1	38 (24.1)	29 (24.4)	5 (33.3)	4 (16.7)
N2	62 (39.2)	46 (38.7)	5 (33.3)	11 (45.8)
**M stage**				
M0	158 (100)	119 (100)	15 (100.0)	24 (100.0)
M1	0 (0)	0 (0)	0 (0)	0 (0)
**AJCC stage**				
III	63 (39.9)	50 (42.0)	9 (60.0)	4 (16.7)
IV	95 (60.1)	69 (58.0)	6 (40.0)	20 (83.3)
**Recurrence**				
Yes	41 (25.9)	30 (25.2)	6 (40.0)	5 (20.8)
No	117 (74.1)	89 (74.8)	9 (60.0)	19 (79.2)
**Metastasis**				
Yes	4 (2.5)	2 (1.7)	1 (6.7)	1 (4.2)
No	154 (97.5)	117 (98.3)	14 (93.3)	23 (95.8)
**Adjuvant RT/CRT**				
Yes	111 (70.3)	81 (68.1)	11 (73.3)	19 (79.2)
No	47 (29.7)	38 (31.9)	4 (26.7)	5 (20.8)

NAC, neoadjuvant chemotherapy; NAICT, neoadjuvant immunochemotherapy; R0, negative margins; AJCC, American Joint Committee on Cancer; RT, radiotherapy; CRT, chemoradiotherapy.

### 3.2 Prognostic factors for OS and DFS

Differentiation, negative margins (R0), N stage, recurrence and postoperative metastasis were significantly related to 3-year OS. Similarly, differentiation, negative margins (R0), N stage, treatment regimens, recurrence and postoperative metastasis were significantly related to 3-year DFS (Table 2).

**Table 2:** Log-rank test results for OS and DFS.

Characteristics	OS	DFS
	*P* value	*P* value
Age	0.100	0.050
Sex	0.239	0.319
Smoking history	0.642	0.995
Alcohol history	0.239	0.110
Tumor location	0.896	0.824
Differentiation	0.004	0.019
R0	0.001	0.003
T stage	0.165	0.051
N stage	0.011	0.048
AJCC stage	0.198	0.374
Treatment	0.056	0.004
Recurrence	<0.001	<0.001
Postoperative metastasis	0.042	<0.001

OS, overall survival; DFS, disease-free survival; R0, negative margins; AJCC, American Joint Committee on Cancer.

The univariate and multivariate analysis showed that, there were 6 independent prognostic factors for 3-year OS, including differentiation, negative margins (R0), N stage, treatment regimens, recurrence and postoperative metastasis. Among them, low differentiation (*P* = 0.015; HR, 4.963; 95% CI, 1.362–18.088), recurrence (*P* < 0.001; HR, 2.951; 95% CI, 1.741–5.004) and postoperative metastasis (*P* = 0.044; HR, 3.614; 95% CI, 1.032–12.654) were all risk factors. Negative margins (*P* = 0.043; HR, 0.381; 95% CI, 0.150–0.969) and NAICT followed by surgery (*P* = 0.004; HR, 0.213; 95% CI, 0.074–0.614) were confirmed to be protective factors for patients (Table 3).

**Table 3:** Univariate and multivariate analysis results for OS.

Characteristics	Univariate Analysis HR (95% CI)	*P* value	Multivariate Analysis HR (95% CI)	*P* value
**Age (years)**		0.154		
81–90	-	-		
≤40	0.191 (0.035,1.044)	0.056		
41–50	0.287 (0.088,0.935)	0.038		
51–60	0.279 (0.095,0.822)	0.021		
61–70	0.321 (0.110,0.936)	0.037		
71–80	0.184 (0.054,0.631)	0.007		
**Sex**				
Male	-	-		
Female	0.722 (0.417,1.250)	0.245		
**Smoking history**				
No	-	-		
Yes	0.886 (0.529,1.483)	0.645		
**Alcohol history**				
No	-	-		
Yes	1.366 (0.807,2.313)	0.245		
**Tumor location**		0.902		
Tongue	-	-		
Hard palate	0.580 (0.139,2.415)	0.454		
Gum	0.815 (0.361,1.838)	0.621		
Floor of the mouth	1.105 (0.511,2.386)	0.800		
Cheek mucosa	0.716 (0.343,1.493)	0.373		
Lip	0.898 (0.216,3.742)	0.883		
**Differentiation**		0.008	-	0.041
High	-	-	-	-
Middle	3.412 (1.060,10.983)	0.040	2.931 (0.895,9.595)	0.076
Low	6.474 (1.846,22.425)	0.003	4.963 (1.362,18.088)	0.015
**R0**				
No	-	-	-	-
Yes	0.256 (0.109,0.600)	0.002	0.381 (0.150,0.969)	0.043
**T stage**		0.185		
T1	-	-		
T2	1.638 (0.370,7.260)	0.516		
T3	2.911 (0.696,12.187)	0.143		
T4	2.008 (0.464,8.694)	0.351		
**N stage**		0.015	-	0.046
N0	-	-	-	-
N1	1.377 (0.672,2.821)	0.383	0.909 (0.429,1.925)	0.803
N2	2.346 (1.286,4.280)	0.005	1.866 (0.987,3.528)	0.055
**AJCC stage**				
III	-	-		
IV	1.407 (0.831,2.380)	0.204		
**Treatment**		0.079	-	0.016
Surgery	-	-	-	-
NAC + surgery	1.110 (0.504,2.446)	0.795	0.854 (0.381,1.914)	0.701
NAICT + surgery	0.318 (0.115,0.881)	0.028	0.213 (0.074,0.614)	0.004
**Recurrence**				
No	-	-	-	-
Yes	3.489 (2.133,5.759)	<0.001	2.951 (1.741,5.004)	<0.001
**Metastasis**				
No	-	-	-	-
Yes	3.112 (1.005,9.958)	0.049	3.614 (1.032,12.654)	0.044

OS, overall survival; NAC, neoadjuvant chemotherapy; NAICT, neoadjuvant immunochemotherapy; R0, negative margins; AJCC, American Joint Committee on Cancer.

The univariate and multivariate analysis showed that, there were 5 independent prognostic factors for 3-year DFS, including negative margins (R0), N stage, treatment regimens, recurrence and postoperative metastasis. Among them, N2 stage (*P* = 0.004; HR, 2.752; 95% CI, 1.390–5.451), recurrence (*P* < 0.001; HR, 4.979; 95% CI, 2.856–8.681) and postoperative metastasis (*P* < 0.001; HR, 51.317; 95% CI, 10.830–243.162) were all risk factors. Negative margins (*P* = 0.041; HR, 0.326; 95% CI, 0.111–0.956) and NAICT followed by surgery (*P* = 0.006; HR, 0.219; 95% CI, 0.074–0.650) were confirmed to be protective factors for patients (Table 4).

**Table 4:** Univariate and multivariate analysis results for DFS.

Characteristics	Univariate Analysis HR (95% CI)	*P* value	Multivariate Analysis HR (95% CI)	*P* value
**Age (years)**		0.092		0.095
81–90	-	-	-	-
≤40	0.148 (0.027,0.809)	0.028	0.402 (0.063,2.543)	0.333
41–50	0.329 (0.106,1.024)	0.055	0.621 (0.169,2.276)	0.472
51–60	0.256 (0.088,0.747)	0.013	0.444 (0.137,1.445)	0.178
61–70	0.247 (0.084,0.723)	0.011	0.527 (0.152,1.827)	0.312
71–80	0.186 (0.057,0.606)	0.005	0.142 (0.035,0.581)	0.007
**Sex**				
Male	-	-		
Female	0.776 (0.469,1.286)	0.325		
**Smoking history**				
No	-	-		
Yes	0.998 (0.617,1.616)	0.995		
**Alcohol history**				
No	-	-		
Yes	1.485 (0.906,2.434)	0.116		
**Tumor location**		0.836		
Tongue	-	-		
Hard palate	0.499 (0.121,2.067)	0.338		
Gum	0.678 (0.304,1.515)	0.344		
Floor of the mouth	0.863 (0.404,1.843)	0.703		
Cheek mucosa	0.758 (0.389,1.478)	0.416		
Lip	0.720 (0.174,2.981)	0.650		
**Differentiation**		0.027		0.385
High	-	-	-	-
Middle	2.417 (0.964,6.059)	0.060	2.050 (0.738,5.696)	0.168
Low	3.970 (1.427,11.043)	0.008	1.831 (0.585,5.725)	0.298
**R0**				
No	-	-	-	-
Yes	0.306 (0.132,0.713)	0.006	0.326 (0.111,0.956)	0.041
**T stage**		0.062		0.619
T1	-	-	-	-
T2	1.213 (0.349,4.223)	0.761	0.878 (0.226,3.414)	0.851
T3	2.468 (0.758,8.028)	0.133	1.424 (0.366,5.543)	0.611
T4	1.482 (0.436,5.030)	0.529	1.425 (0.361,5.615)	0.613
**N stage**		0.056		0.003
N0	-	-	-	-
N1	1.423 (0.745,2.717)	0.285	1.233 (0.523,2.908)	0.632
N2	1.969 (1.129,3.433)	0.017	2.752 (1.390,5.451)	0.004
**AJCC stage**				
III	-	-		
IV	1.242 (0.765,2.018)	0.380		
**Treatment**		0.008		0.004
Surgery	-	-	-	-
NAC + surgery	1.861 (0.948,3.652)	0.071	1.971 (0.914,4.247)	0.083
NAICT + surgery	0.296 (0.107,0.817)	0.019	0.219 (0.074,0.650)	0.006
**Recurrence**				
No	-	-	-	-
Yes	5.667 (3.499,9.178)	<0.001	4.979 (2.856,8.681)	<0.001
**Metastasis**				
No	-	-	-	-
Yes	31.762 (10.089,99.996)	<0.001	51.317 (10.830,243.162)	<0.001

DFS, disease-free survival; NAC, neoadjuvant chemotherapy; NAICT, neoadjuvant immunochemotherapy; R0, negative margins; AJCC, American Joint Committee on Cancer.

### 3.3 Survival

The median follow-up time was 36.0 months. The 3-year restricted mean survival time (RMST) for OS among all patients was 27.31 months (95% CI, 25.42–39.19). For patients receiving surgery alone, the 3-year OS RMST was 26.29 months (95% CI, 24.04–28.53). Among those treated with NAC followed by surgery, the 3-year OS RMST was 25.80 months (95% CI, 19.41–32.19), while patients undergoing NAICT followed by surgery achieved a 3-year OS RMST of 33.29 months (95% CI, 30.62–35.97).

Regarding DFS, the cohort's 3-year RMST was 25.04 months (95% CI, 22.94–27.13). The surgery alone group showed a 3-year DFS RMST of 24.56 months (95% CI, 22.16–26.97). Patients received NAC plus surgery had a 3-year DFS RMST of 17.73 months (95% CI, 10.79–24.68), whereas the NAICT plus surgery group demonstrated a 3-year DFS RMST of 31.96 months (95% CI, 28.00–35.91).

**Figure 2:**
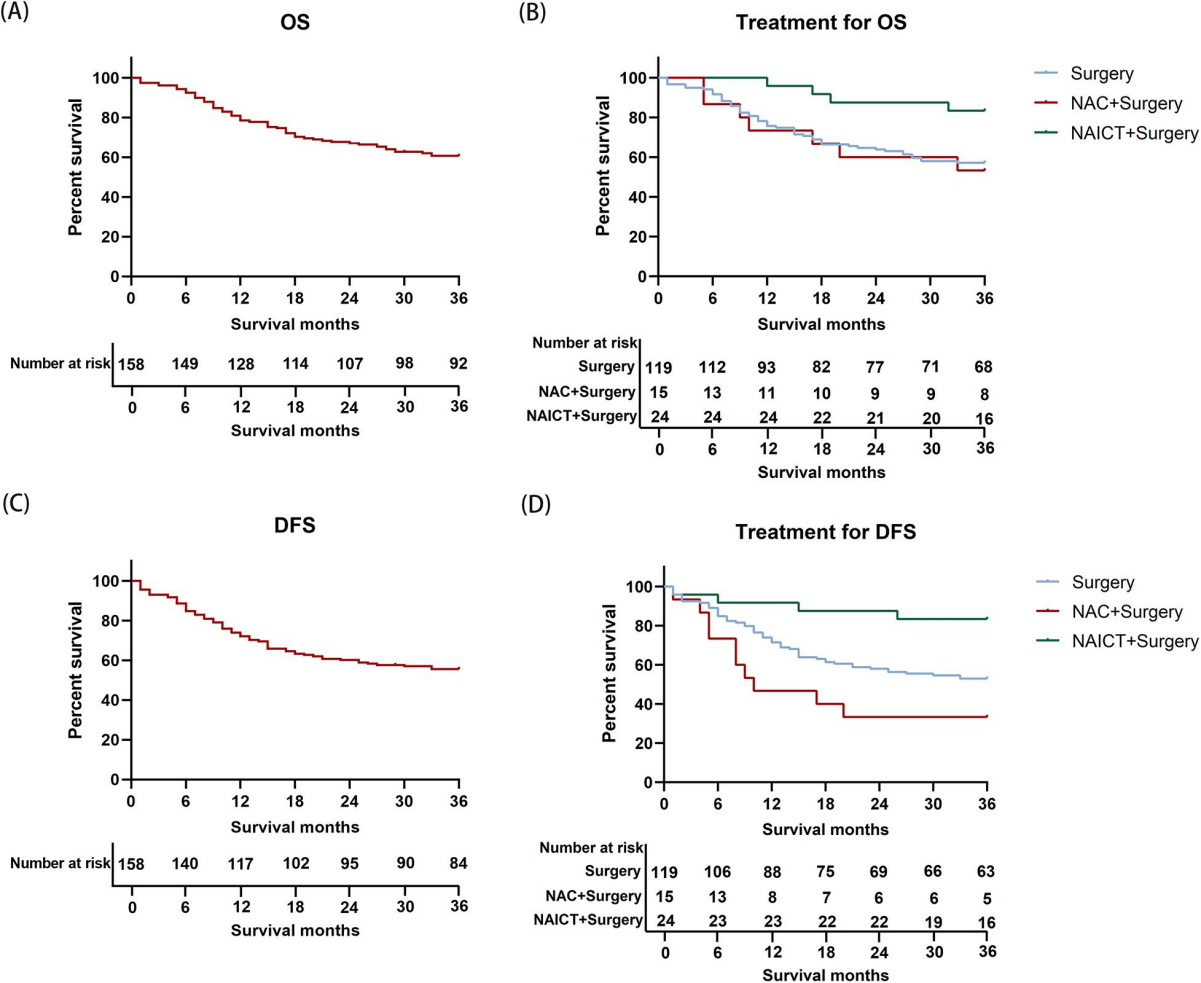
The prognosis of enrolled patients. **(A)** Overall survival of the enrolled patients. **(B)** Overall survival of different treatment regimens. **(C)** Disease-free survival of the enrolled patients. **(D)** Disease-free survival of different treatment regimens.

Compared to direct surgery, surgery following NAC showed no significant difference in 3-year OS (53.3% vs. 57.1%, *P* = 0.701) and 3-year DFS (33.3% vs. 52.9%, *P* = 0.083). Compared to direct surgery, preoperative NAICT significantly improved 3-year OS (82.4% vs. 57.1%, *P* = 0.004) and 3-year DFS (83.3% vs. 52.9%, *P* = 0.006). The study conclusions were further supported by the propensity score-weighted IPTW analysis (*P* < 0.001), which reinforced the reliability and stability of the results (Table 5). Furthermore, compared to surgery following NAC, preoperative NAICT was associated with a significantly improved 3-year OS (82.4% vs. 53.3%, *P* = 0.03) and 3-year DFS (83.3% vs. 33.3%, *P* = 0.001) (Figure 2).

**Table 5:** Multivariate analysis and propensity score-weighted IPTW analysis for OS and DFS.

Characteristics	Multivariate Analysis HR (95% CI)	*P* value	IPTW Analysis HR (95% CI)	*P* value
**OS**	-	-	-	-
Surgery	-	-	-	-
NAC + surgery	0.854 (0.381,1.914)	0.701	0.984 (0.387,2.495)	0.972
NAICT + surgery	0.213 (0.074,0.614)	0.004	0.151 (0.060,0.379)	<0.001
**DFS**	-	-	-	-
Surgery	-	-	-	-
NAC + surgery	1.971 (0.914,4.247)	0.083	1.778 (0.833,3.795)	0.137
NAICT + surgery	0.219 (0.074,0.650)	0.006	0.155 (0.066,0.362)	<0.001

OS, overall survival; DFS, disease-free survival; IPTW, inverse probability of treatment weights; NAC, neoadjuvant chemotherapy; NAICT, neoadjuvant immunochemotherapy.

### 3.4 Pathological and radiological response to preoperative NAICT

**Figure 3:**
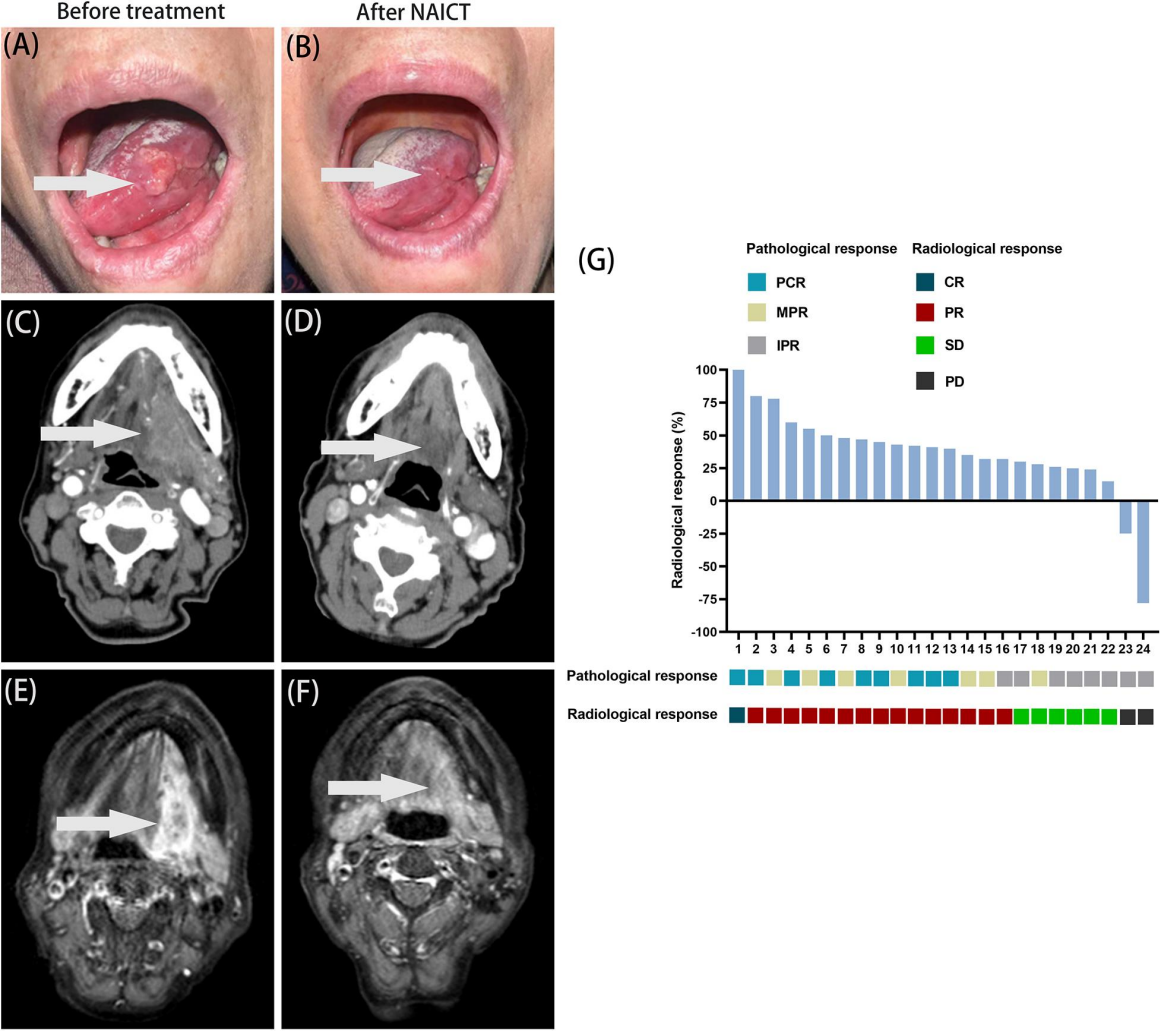
Evaluation of the efficacy of NAICT. **(A–F)** A 75-year-old female patient with carcinoma of the tongue was diagnosed at a tumor stage of pT3N1M0. **(A)**, **(C)**, **(E)** The primary lesion of tumor, computed tomography and magnetic resonance imaging before treatment. **(B)**, **(D)**, **(F)** The primary lesion of tumor, computed tomography and magnetic resonance imaging after NAICT. **(G)** The patient's treatment response following NAICT (*N* = 24). NAICT, neoadjuvant immunochemotherapy; PCR, pathological complete response; MPR, major pathologic response; IPR, incomplete pathologic response; CR, complete response; PR, partial response; SD, stable disease; PD, progressive disease. Horizontal axis: case number.

Figure 3 illustrates the pathological and radiological evaluation of the primary tumor in 24 patients who underwent radical resection of the primary lesion after preoperative NAICT. Among these patients, 9 achieved PCR in primary tumors, 7 attained MPR, and the remaining 8 showed IPR. Regarding imaging assessments, 1 of 24 patients exhibited CR in primary tumors, 15 demonstrated PR, 6 maintained SD, while the other 2 developed PD (Figure 3).

### 3.5 Safety assessment of preoperative NAICT

Among the 24 patients, 17 (70.8%) experienced treatment-related adverse events, all of which were grade 1 or 2. Anemia (*n* = 15, 62.5%) was the most common hematological adverse effects, followed by Alopecia (*n* = 14, 58.3%) and Thyroid dysfunction (*n* = 13, 54.1%). All the adverse effects were relieved after the corresponding treatment (Table 6).

**Table 6:** The treatment-related adverse effects of NAICT.

Toxicity	Grade 1 (%)	Grade 2 (%)	Grade 3 (%)	Grade 4 (%)
Leukopenia	7 (29.2)	5 (20.8)	0	0
Neutropenia	7 (29.2)	5 (20.8)	0	0
Anemia	10 (41.7)	5 (20.8)	0	0
Thrombocytopenia	7 (29.2)	0	0	0
Thyroid dysfunction	5 (20.8)	8 (33.3)	0	0
Rash	2 (8.3)	0	0	0
Fatigue	7 (29.2)	0	0	0
Nausea	7 (29.2)	5 (20.8)	0	0
Diarrhea	1 (4.2)	0	0	0
Alopecia	7 (29.2)	7 (29.2)	0	0
Hepatotoxicity	1 (4.2)	0	0	0
Nephrotoxicity	0	2 (8.3)	0	0

NAICT, neoadjuvant immunochemotherapy.

## 4 Discussion

This study aimed to determine whether NAICT administered prior to surgery influences the prognosis of locally advanced OSCC. Through a retrospective cohort study at our institution, we demonstrated that preoperative NAICT may have a more favorable impact on survival outcomes in patients with locally advanced OSCC. Furthermore, 66.6% of patients in the NAICT cohort achieved MPR, with tolerable adverse reactions during the treatment period.

In this investigation, the 3-year OS and DFS in the group receiving NAC followed by surgery were not significantly different from those in the direct surgery group. These findings were in concordance with the outcomes by Zhong et al. (25). Their study enrolled 256 patients with locally advanced OSCC. The experimental group received two cycles of TPF regimen (docetaxel, cisplatin, and 5-fluorouracil) neoadjuvant chemotherapy followed by surgery, while the control group underwent surgery directly. The results demonstrated no significant difference in 2-year OS (68.2% vs 68.8%) or DFS (63.6% vs 62.2%) between patients receiving preoperative NAC and those undergoing direct surgery. These findings suggested that preoperative NAC does not significantly improve the prognosis of patients with locally advanced OSCC. In subgroup analysis (25), patients who achieved clinical and pathological responses after chemotherapy exhibited a more favorable prognosis. This phenomenon indicated that the sensitivity of OSCC to chemotherapy is heterogeneous. Therefore, identifying patients sensitive to chemotherapy in future research will be crucial for developing personalized induction therapy strategies for OSCC.

In recent years, immunotherapy, particularly ICIs, has achieved remarkable success in recurrent and metastatic HNSCC (26). Immunotherapy, whether administered as a monotherapy or in conjunction with chemotherapy, has consistently exhibited enhanced efficacy when contrasted with the conventional targeted therapy paired with chemotherapy (27). Reportedly, immunotherapy monotherapy elicits a relatively low MPR rate in OSCC (6% for pembrolizumab and 8% for nivolumab) (28–30). Preclinical evidence indicated that the combination of chemotherapy with PD-1 inhibitor treatment could potentially augment antigen presentation due to tumor cell apoptosis, thus stimulated T cell-mediated immune responses. Consequently, NAICT holds significant potential for treating OSCC. In this study, NAICT achieved a MPR rate of 66.6%. Achieving MPR following neoadjuvant therapy has been validated as a predictor for longer survival (31). During our subsequent follow-up, the 3-year OS and DFS rates in the NAICT group were significantly higher than that in the surgery-alone group and surgery following NAC group, indicating that NAICT could be a more suitable approach for controlling locoregional recurrence and metastasis, thereby improving the overall prognosis in locally advanced OSCC patients. These findings were consistent with previous research. Huang et al. reported a 60% MPR rate with NAICT, alongside 2-year progression-free survival and OS rates of 90% and 95%, respectively (32). Similarly, Li et al. reported the 3-year estimated DFS rates were 89.0% in the NAICT cohort compared to 60.8% in the non-NAICT cohort, while the 3-year estimated OS rates were 91.3 vs. 64.0%, respectively (33). Our study, for the first time, compares the medium-term prognostic impact of three distinct treatment strategies for locally advanced OSCC and demonstrates that preoperative NAICT may have a more favorable impact on the prognosis compared to both surgery alone and surgery following NAC. Recently, the KEYNOTE-689 phase III trial reported that event-free survival at 36 months was 57.6% in the pembrolizumab group and 46.4% in the control group (surgery and adjuvant radiotherapy with or without concomitant cisplatin) among participants with locally advanced HNSCC (22). Therefore, NAICT demonstrates considerable potential in HNSCC treatment, and this treatment modality may reshape clinical guidelines and warrants further investigation.

It was considerable to mention that two individuals in the preoperative NAICT cohort experienced PD during the immunochemotherapy phase. Both cases subsequently developed recurrence after surgery, and one died during the follow-up period. Therefore, early identification of patients at risk of developing PD during immunochemotherapy is crucial. Research in breast cancer has reported associations between PD during chemotherapy and factors such as race, tumor stage, Ki-67 score, and Tumor-Infiltrating Lymphocytes (TILs) (34, 35). Currently, predictive factors for PD during neoadjuvant therapy in patients with OSCC remain unclear. The limited sample size employed in this investigation precludes statistically conclusive findings. It is imperative that subsequent studies concentrate on identifying cases that experience PD during neoadjuvant therapy.

In this study, treatment-related adverse events occurred in 70.8% of patients receiving immunochemotherapy. All events were grade 1–2 in severity and resolved following treatment. The incidence of grade 3–4 NAICT-related adverse events reported in previous studies ranges from 0% to 15%, which is consistent with the findings of our study (32, 33, 36, 37), suggesting that the NAICT regimen has a manageable toxicity profile, although careful monitoring and supportive care remain imperative. Furthermore, consistent with previous studies, no surgery was postponed due to NAICT-related adverse events, which further supports the favorable tolerability and feasibility of this regimen in patients with locally advanced OSCC (33).

This study has several limitations. First, as a retrospective study, biases are inevitably present. While several strategies were adopted to minimize their influence—including the application of strict inclusion and exclusion criteria, a high follow-up rate, independent review of imaging by multiple assessors, and statistical adjustment—the findings should still be interpreted with appropriate caution. Second, the cohort of patients receiving neoadjuvant therapy was relatively small, despite the propensity score-weighted IPTW analysis was conducted to enhance comparability between groups. Future investigations would benefit from more rigorous designs, such as adequately powered randomized controlled trials, to further validate these results. Third, NAICT was initiated at our institution around 2021, precluding assessment of 5-year OS or DFS at this time. We are continuing to follow these patients and will report the longer-term prognostic outcomes of NAICT in future studies.

## 5 Conclusion

Compared to direct surgery or NAC followed by surgery, the multimodal treatment approach of surgery after NAICT demonstrated a more favorable impact on the prognosis of patients with locally advanced OSCC, while exhibiting manageable treatment-related adverse effects. In summary, the NAICT regimen demonstrates both efficacy and an acceptable safety profile in the treatment of locally advanced OSCC, supporting its broader clinical implementation.

## Data Availability

The raw data supporting the conclusions of this article will be made available by the authors, without undue reservation.
